# Editorial: Preventing Developmental Brain Injury—From Animal Models to Clinical Trials

**DOI:** 10.3389/fneur.2019.00775

**Published:** 2019-07-19

**Authors:** Masahiro Tsuji, Stéphane V. Sizonenko, Olivier Baud

**Affiliations:** ^1^Department of Food and Nutrition, Kyoto Women's University, Kyoto, Japan; ^2^Division of Development and Growth, Department of Pediatrics, Gynecology and Obstetrics, School of Medicine, University of Geneva, Geneva, Switzerland; ^3^Division of Neonatology, Department of Pediatrics Gynecology and Obstetrics, School of Medicine, University of Geneva, Geneva, Switzerland; ^4^Robert Debré Hospital, INSERM U1141, Paris-Diderot University, Paris, France

**Keywords:** neonatal encephalopathy, brain injury, prematurity and low birth weight, animal model, clinical study

A variety of brain injuries originating from fetal, perinatal, and neonatal periods may cause mortality, and many of the survivors of those injuries suffer from life-long morbidity. It is our task as researchers to prevent and reduce such brain injuries in infants. This is why we Topic Editors chose the theme “Preventing developmental brain injury—from animal models to clinical trials.”

Among those developing brain injuries, neonatal encephalopathy in full-term infants has been the most crucial issue for decades (1). Neonatal encephalopathy refers to newborns with symptoms of acute cerebral dysfunction such as depressed consciousness, abnormal muscle tone, respiratory distress, feeding difficulties, and seizures (2, 3). Hypoxic-ischemic injury is the most common (up to 85% of cases), and ischemic/hemorrhagic stroke is the second most common, etiology and pathophysiology of neonatal encephalopathy (3). Approximately 3% of babies born in the world die by 4 weeks of age, and asphyxia, i.e., neonatal encephalopathy accounts for 23% of neonatal mortality (4); hence, the mortality rate due to neonatal encephalopathy is estimated to be 70/1,000 livebirths. Almost all (99%) neonatal mortality occurs in resource-limited countries (4). The incidence of neonatal encephalopathy in term newborns has been declining in resource-rich countries, and is currently 1/1,000 livebirths or less (5, 6). Nevertheless, nearly half of infants with neonatal encephalopathy in resource-rich countries die or develop permanent neurological disabilities, such as cerebral palsy, intellectual disability, and epilepsy (7). Therefore, the burden of neonatal encephalopathy remains high for patients, caregivers, and society.

Currently, the most common brain injuries caused during fetal, perinatal, and neonatal periods are those associated with prematurity, i.e., fetal growth restriction, preterm birth, and low birth weight (8). Approximately 15 million newborns were estimated to be born preterm (<37 weeks of gestation) in 2010, which accounted for 11% of all livebirths worldwide, ranging from 5% in some European countries to 18% in some African countries (9). The preterm birth rate has been increasing over the past two decades (9). Similarly, the rate of low birth weight (birth weight is <2,500 g) has been increasing: 7–9% of all live births in resource-rich countries (10, 11) and 11–12% in resource-limited countries (12). Intrauterine hypoperfusion and inflammation are the two leading causes of brain injuries associated with prematurity (13–16). Although most infants do not present obvious neurological symptoms during the neonatal period, they may develop cerebral palsy and neurodevelopmental disorders such as attention-deficit/hyperactive disorders and autistic spectrum disorders during childhood (17, 18). Survivors of very low birth weight (<1,500 g) may present cerebral palsy in 5–10% of the individuals and cognitive/behavioral/attentional deficits in ~50% of these individuals (19, 20). The main focus of outcome studies in neonatal care has been the extremely low-birth-weight (<1,000 g) infants and extremely preterm infants [born before 28 weeks of gestation (9)]. Recent studies, however, have shown that even infants born with mild prematurity (birth weight is 1,500– <2,500 g; gestational age is 32– <37 weeks) have significantly higher risks of developing numerous neurological, psychological, and general health problems later in life (21). This phenomenon is now widely known as DOHaD (developmental origin of health and diseases) (22). While many preclinical studies have been conducted in neonatal encephalopathy, a limited number of preclinical studies have been conducted regarding brain injuries associated with prematurity.

Neonatal encephalopathy and brain injury associated with prematurity seem to be contrasting problems, as the former is a medical emergency during the neonatal period, while the latter is a gradually emerging problem in childhood. While it is important to shed light on rare brain injuries, neonatal encephalopathy in term infants and brain injuries associated with prematurity are the most prominent issues that researchers and funding sources need to address considering their impact on patients and society.

This Research Topic collects 29 articles: 20 animal studies (including 1 *in vitro* study), 3 clinical studies, 1 combined study with clinical and animal studies, and 5 reviews. Of the 21 animal studies, the majority used rodents, and 3 studies used large animals, i.e., piglets or lambs. Hypoxic-ischemic injury was the most frequently used model (11 articles) in this Research Topic collection. Other models included intrauterine ischemia/inflammation (3 articles) and irradiation (2 articles). As the theme of this Research Topic was “preventing developing brain injury,” many of the animal studies explored the effects of novel therapies. Five studies examined the effects of cell-based therapies, 2 studies examined erythropoietin, and others examined melatonin, inhaled carbon dioxide therapy, and protective ventilation strategies. Some studies examined the effects of certain genomic modifications as possible targets of novel therapies. Clinical studies investigated EEG and blood pressure, IL-10 gene polymorphism, and cerebral lactate in infants with brain damage. Despite the Topic Editors' expectations, there was no manuscript submission of an interventional clinical trial, which may suggest a scarcity of clinical trials and difficulty in conducting clinical trials of novel therapies in sick infants. The review articles summarized the effects of therapies, such as oxytocin, magnesium, and nitric oxide synthase inhibition.

With respect to the demographics of this Research Topic collection, the corresponding author's laboratories are located in a variety of countries: Japan (6 articles), the United States (5 articles), France (4 articles), the United Kingdom (3 articles), Sweden (3 articles), the Netherlands (2 articles), Switzerland (1 article), Germany (1 article), China (1 article), South Korea (1 article), Canada (1 article), and Australia (1 article). Thirteen articles represent international collaborative studies.

The Topic Editors are pleased to have a wide variety of 29 articles from many countries around the world. None of the articles in this collection, however, are from the resource-limited world. It is important to encourage research in this area in resource-limited countries where developmental brain injuries are expected to be most prevalent. We hope that research on neonatal brain injuries will become even more active and will contribute to better preventions and treatments for these injuries in the near future.

## Author Contributions

MT wrote the draft. SS and OB critically reviewed the manuscript.

### Conflict of Interest Statement

The authors declare that the research was conducted in the absence of any commercial or financial relationships that could be construed as a potential conflict of interest.
